# Management of Single-Rooted Maxillary Central Incisor with Two Canals: A Case Report

**Published:** 2012-03-01

**Authors:** Anantanarayanan Krishnamurti, Natanasabapathy Velmurugan, Suresh Nandini

**Affiliations:** 1. Department of Conservative Dentistry and Endodontics, Meenakshi Ammal Dental College, Tamilnadu, India

**Keywords:** Maxillary Central Incisor, Root Canal, Root Resorption, Tooth Root

## Abstract

The aim of this study was to report the endodontic management of right maxillary central incisor having two canals with type IV Vertucci canal configuration. Internal morphology of root canals is variable and often complex. Therefore, to achieve a technically satisfactory endodontic outcome, the clinician must have adequate knowledge of the internal canal morphology and its variations in order to debride and obturate the root canal system thoroughly.

## Introduction

The success of endodontic treatment closely depends on complete knowledge of the complexity and variety of internal/external dental anatomy in order to identify, clean, shape and obturate the whole root canals. Maxillary central incisor is considered to be the least difficult tooth for RCT. It is generally considered as tooth with a single root and single root canal [1]. However, the internal anatomy of the tooth can present a number of variations; these are extremely rare and in most cases are associated with anomalous tooth development such as gemination, fusion, dens invaginatus or presence of supernumerary root [2]. The incidence of an additional canal in the maxillary central incisor is ≈0.6% [3]. This case report highlights the clinical significance and management of a rare case of maxillary central incisor having two canals with vertucci canal pattern type IV [4].

## Case report

A 29-year-old female patient with the chief complaint of intermittent dull pain in right maxillary central incisor was referred for RCT. Her medical history was non-contributory. She had undergone orthodontic treatment during last three years. On clinical examination, right maxillary central incisor did not exhibit any morphological variation. There was absence of dental caries, periodontal probing and mobility was within physiologic limits. Generalized spacing was present between the dentition and right maxillary incisor exhibited a mild distal rotation. The tooth did not respond to both electric (Parkell Electronics, Farmingdale, USA) and thermal pulp testing. Hence a provisional diagnosis of pulpal necrosis was made.

**Figure 1:**
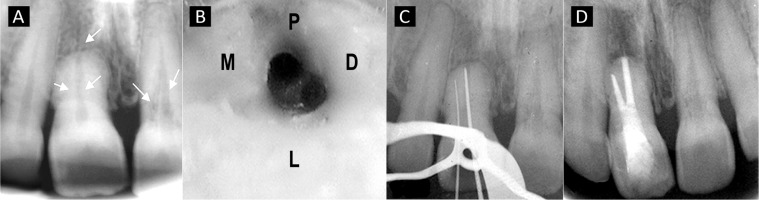
A) Preoperative radiograph showing the presence of additional canal in both 11 and 21; B)11Disto-labial position of additional canal to main canal under 3X magnification; C) Working length radiograph showing presence of 2 separate canals in; D) Post-obturation radiograph of 11

Preoperative radiograph revealed the presence of a single root with external apical root resorption in right maxillary central incisor with receded pulp chamber (Figure 1A). A faint radiolucent line was observed in both the right and left central incisors in addition to the main canal on the radiograph. Hence presence of an additional root canal in both the incisors was suspected. Multiple angulated radiographs were taken to confirm the presence of extra canals.

Access opening was performed under rubber dam isolation. The main canal was located and the access was further modified to locate the additional canal. Using a surgical operating microscope (Seiler Revelation, St. Louis, MO, USA) under ×3 magnification the additional canal was located distolabial to the main canal (Figure 1B).

Radiographic working length was determined using ISO 0.02 taper K-files (Figure 1C ). The orifice was enlarged to #3 Gates Glidden drill (Dentsply, Ballaigues, Switzerland) in the main canal and with #2 in the additional canal. The canal was cleaned and shaped with K-files (Dentsply, Ballaigues, Switzerland) using step back technique. Canals were irrigated with 2.5% NaOCl, 17% EDTA (Prime Dental Products Pvt. Ltd, India) and saline. Calcium hydroxide (Vitapex, J. Morita, Tokyo, Japan) was placed as an intracanal medicament using lentulo spiral (Dentsply, Ballaigues, Switzerland) and the access was sealed with Cavit (ESPE America, Norristown, PA, USA).

The patient was recalled after three weeks and was found to be asymptomatic. The medicament was changed and the patient was kept under observation for three more weeks. In the subsequent visit, calcium hydroxide was retrieved using ultrasonics (Satelec, P5XS, Aceton euipments, NA, USA) and the canals were rinsed with saline and dried using absorbent paper points. Canals were Obturated with gutta- percha (Dentsply Ballaigues, Switzerland) using cold lateral compaction technique with AH plus resin sealer (Maillefer, Konstanz, Germany) and access cavity was restored with composite resin Filtek Z250 (3M Dental Products, St Paul, MN, USA). Patient was asymptomatic during the 1year follow-up period (Figure 1D ).

## Discussion

The root canal geometry may have a direct impact on the thoroughness and extent of debridement and root canal shaping. Such morphological variations are attributed to the disturbances in the normal development of Hertwig’s epithelial root sheath and may adversely affect the outcome of endodontics. Literature reveals that the canal variations of maxillary central incisor include the presence of two/three canals mostly associated with gemination, fusion or supernumerary root (Table 1). Mangani et al. has reported a case of maxillary central incisor with dens invaginatus and four root canals [10]. Sert and Beyrilli reported the presence of an additional canal in 3 of the 200 maxillary central incisor examined using demineralisation (≈ 1.5%;Table 2 ) [20].

**Table 1:** Table summarizing previous case reports of Maxillary central incisors with variations in canal morphology

**Author**	**Year**	**Canals (n)**	**Roots (n)**	**Special findings**
**Mader and Konzelman [5]**	1980	2	2	
**Sinai *et al. *[6]**	1980	2	2	
**Hososmi *et al. *[7]**	1989	3	2	Gemination
**Al-Nazhan [8]**	1991	2	2	Enamel Hypoplasia
**Lambruschini and Camps [9]**	1993	2	2	
**Mangani and Ruddle [10]**	1994	4	1	Dens invaginatus
**Cabo-Valle [11]**	1999	2	2	
**Cimilli H and Kartal [12]**	2002	2	2	Fusion of roots
**Genovese and Marsico [13]**	2003	2	2	
**Khojastehpour and Khaya [14]**	2005	2	2	
**Lin *et al. *[15]**	2006	2	2	
**Sponchiado *et al. *[16]**	2006	2	2	
**Benenati [17]**	2006	2	2	
**Mahshid Sheikh [18]**	2007	3	1	
**Gondim E Jr* et al.* [19]**	2009	3	2	
**Present case**	2012	2	1	

**Table 2:** Table summarizing the Demineralization studies on maxillary central incisors

**Investigator**	**Report type**	**Examined Teeth (n)**	**Incidence**	
			**Type II**	**Type IV**
Vertucci [4]	Demineralization and Staining	100	0	0
Sert and Bayirli [20]	Demineralization and Staining	200	1	2
Weng et al. [21]	Modified canal staining	71	3	0

The use of multiple angulated radiographs usually aid in discovery of addition canals [22].

This is dependent on the amount of separation between the canals and is reported to lie between 20º and 40º [23].

**Figure 2:**
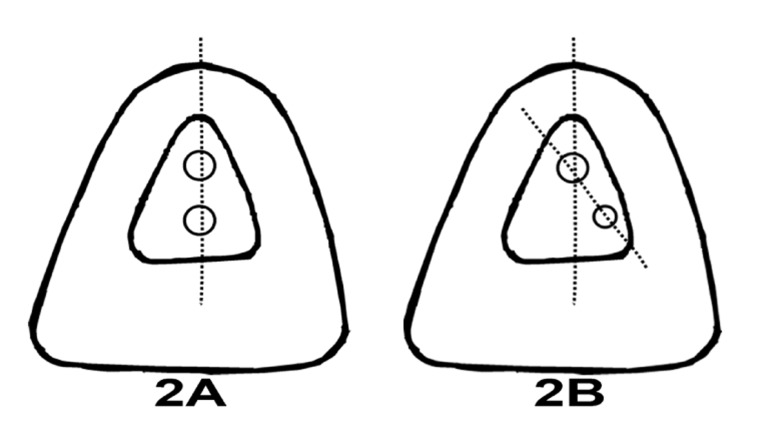
Illustration of the position of additional canal; A) Normal position additional canal to main canal; B) Distolabial position of the additional canal to main orifice

An important feature in this case is the aberration in the position of the additional canal in relation to the main canal orifice. Most reports have so far have documented the presence of additional canal in the same plane as the main canal (Figure 2A). However, the additional canal was present distolabial to the main canal (Figure 1C, Figure 2B). Hence, an extension of the access was required to identify the additional canal. Moreover, the diameter of the additional canal was smaller in comparison to the main canal.

**Figure 3:**
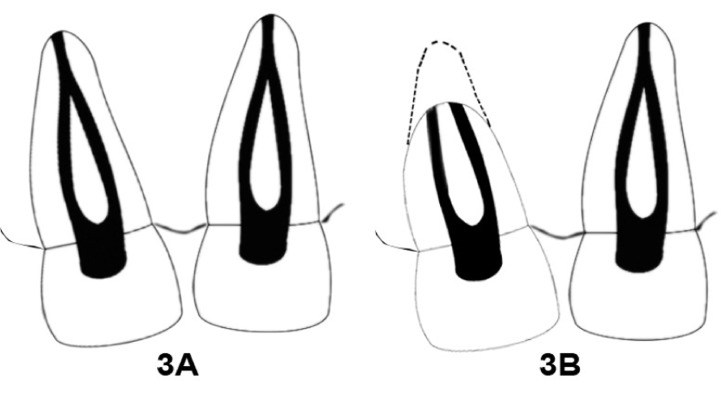
Diagrammatic representation illustrating the change in canal pattern due to resorption0; A) assuming type II canal in 11 prior to resorption; B) assuming the transformation of type II to type IV canal pattern in 11 as a result of resorption

Another interesting feature was the difference in the canal pattern observed on the demineralisation in 11 and 21. Sabala et al. reported that bilateral symmetry is a feature of aberrant anatomy and the rarer the aberration, the more common is the bilateral symmetry [24]. Type IV Vertucci pattern was present in right maxillary central incisor, whereas type II canal pattern was observed in left maxillary central incisors on the radiograph with the additional canal joining the main canal in the middle third of the root. It may be assumed that the canal pattern of 11 could also have been type II prior to resorption (Figure 3A) but the progression of external apical root resorption to the mid root level might have resulted in separate exiting of the canals at the resorbed apex thus mimicking a type IV canal pattern in 11 (Figure 3B). Closer interpretation of the radiograph also shows that the angle at which the extra canal originates at the CEJ is not similar in 11 and 21. This probably could be due to the distal rotation of 11. Although 11 after resorption reveals type IV Vertucci canal pattern, its canal course prior to resorption could not be positively deduced.

Presence of external apical root resorption in right maxillary central incisor (Figure 1A) can be attributed to patient’s orthodontic history. The type of tooth movement, type of orthodontic force, its magnitude and duration of treatment are crucial determinants which influences the severity of root resorption [25]. The incidence of resorption affecting incisors was found to be 28.8% in 5-10 years of follow-up period [26]. Calcium hydroxide has been used as the medicament of choice because of its effective antibacterial properties, favorable influence on the local environment at the resorption site and its ability to act against the clastic cells which aids in healing [27][28].

## Conclusion

This case report increases the awareness of clinicians on aberrations in the root canal morphology of maxillary central incisor and need for additional care to possibly identify and treat such cases. Successful management of this case was attributed to multiple angled radiographs and the use of magnification with surgical operating microscope. Therefore, precise exploration is required prior to root canal treatment in order to avoid missing alternative root canals.
